# Food-Derived Opioid Peptides in Human Health: A Review

**DOI:** 10.3390/ijms21228825

**Published:** 2020-11-21

**Authors:** Akanksha Tyagi, Eric Banan-Mwine Daliri, Fred Kwami Ofosu, Su-Jung Yeon, Deog-Hwan Oh

**Affiliations:** Department of Food Science and Biotechnology, College of Agriculture and Life Sciences, Kangwon National University, Chuncheon 200-701, Korea; akanksha@kangwon.ac.kr (A.T.); ericdaliri@kangwon.ac.kr (E.B.-M.D.); fkofosu17@gmail.com (F.K.O.); sujung0811@gmail.com (S.-J.Y.)

**Keywords:** bioactive peptides, endogenous and exogenous opioid peptides, stress, human health

## Abstract

World Health Organization data suggest that stress, depression, and anxiety have a noticeable prevalence and are becoming some of the most common causes of disability in the Western world. Stress-related disorders are considered to be a challenge for the healthcare system with their great economic and social impact. The knowledge on these conditions is not very clear among many people, as a high proportion of patients do not respond to the currently available medications for targeting the monoaminergic system. In addition, the use of clinical drugs is also associated with various side effects such as vomiting, dizziness, sedation, nausea, constipation, and many more, which prevents their effective use. Therefore, opioid peptides derived from food sources are becoming one of the safe and natural alternatives because of their production from natural sources such as animals and plant proteins. The requirement for screening and considering dietary proteins as a source of bioactive peptides is highlighted to understand their potential roles in stress-related disorders as a part of a diet or as a drug complementing therapeutic prescription. In this review, we discussed current knowledge on opioid endogenous and exogenous peptides concentrating on their production, purification, and related studies. To fully understand their potential in stress-related conditions, either as a drug or as a therapeutic part of a diet prescription, the need to screen more dietary proteins as a source of novel opioid peptides is emphasized.

## 1. Introduction

Unhealthy lifestyle and the consumption of an unhealthy diet have been major causes of non-communicable diseases (NCD) in recent years. According to a World Health Organization report, 350 million people suffer from depression, in which 15–25% are Western-type communities [1]. Along with depression, anxiety, stress, cardiovascular diseases, and high blood pressure are becoming part of the compelling health problems worldwide [2]. For several years, sleep apnea, stress, and anxiety responses are mutual and related to each other. Stress is a response by the body to restore homeostatic balance, and these responses may cause damage or may lead to disease conditions. Several diseases arise as a consequence of stress having co-morbidity with sleep disorders [3], In particular, prolonged or persistent stress contributes to elevated hormones such as cortisol, the “stress hormone”, and decreased serotonin and other neurotransmitters in the brain, including dopamine, which have been correlated with depression. As these chemical systems function properly, they control biological processes such as sleep, appetite, energy, and sex drive, and they enable normal moods and emotions to be expressed. However, if the stress response fails to shut down and reset after a stressful situation has passed, it may lead to depression in susceptible individuals.

Sleep problems lead to issues such as reduction of appetite, a decrease of attention, unstable mood, and fatigue. According to statistics from the Mental Health Services administration (USA), 12 months of major depressive disorder in 2017 was around 13.3% for adolescents and 7.1% for adults [4,5]. It was reported that anxiety and depression disorders are more common in women as compared to men with an approximate 2:1 ratio during women’s reproductive years [4,5]. Furthermore, according to a worldwide survey, 45.7% of individuals with long-term major depressive disorders had a lifetime history of one or more than one anxiety disorder [6]. The importance of understanding stress disorders for health especially considering youth should be overemphasized [7].

Food proteins have long been used as good sources of potent bioactive peptides for preventing, managing, and treating human health [8,9]. Food proteins are biomolecules that are involved in different biological functions for improving human health, and bioactive peptides are encrypted in the sequences of proteins and are released at the time of digestion and play important roles in improving health [10,11]. They may have two or several proteinogenic amino acid groups linked with each other by peptide bonds and are released from their native proteins when fermented or treated with enzymes [12,13,14].

Currently available clinical treatments for stress-related disorders are also associated with various side effects such as vomiting, dizziness, sedation, nausea, and constipation. Therefore, nutraceuticals are becoming a promising target, as they are derived from food naturally and are known as food with medicinal benefits. These foods not only contain basic nutrients but are also rich in probiotics, antioxidants, polyphenols, bioactive compounds, and beneficial fatty acids and are known as functional foods. Their promising therapies and regulatory functions with active compounds may become a powerful tool against synthetic drugs [15], but a lot of studies are still needed to understand their roles. Hence, promoting healthy dietary intake has become a central part of the world health organization’s plan of action to prevent and monitor NCDs [8].

### Opioid Peptides

**Figure 1:**
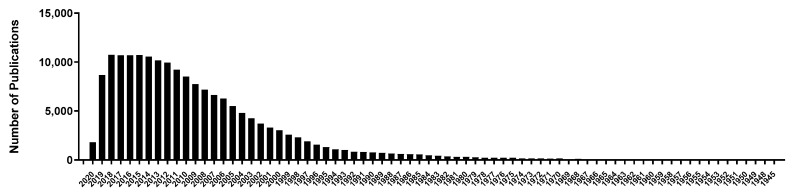
The number of publications on stress-related peptides by year available on PubMed Central as of 30 September 2020.

Opioids were first identified in 1975 and consist of small molecules of 5–80 amino acids [16]. They diffuse locally and act on another adjacent neuron in much lower concentration as compared with neurotransmitters and give a more prolonged response [17]. Opioid peptides bind with opioid receptors μ-, κ-, and δ- for activation [18]. They are known for a wide range of functions such as neuromodulation, sleepiness [19], and pain modulation [20,21]. Except for casexins and lactoferroxins, which are opioid antagonists, the majority of peptide ligands are opioid agonists [22,23]. Opioid peptides can be categorized into endogenous or exogenous peptides based on their origin: endogenous opioid peptides that are produced by the body itself and exogenous opioid peptides that are produced after the ingestion of food when food proteins are digested by body enzymes [24,25,26]. Meanwhile, the number of studies on peptides related to stress is also increasing exponentially, which supports our interest in studying bioactive peptides and their roles in human health (Figure 1). Additionally, several attempts in human, as well as translational animal studies, have confirmed the potential of opioids in stress and related condition. The exponential increase in the search for bioactive peptides from food is also due to their low safety concerns and slow clearance from body tissues compared to synthetic drugs [27].

Here, we will summarize the current literature on opioid peptides, their classification as endogenous and exogenous, production methods, mechanism of action, as well as some opioid-like well-known peptides and their potential roles in stress-related conditions (Table 1).

So far, the potential roles of opioid-like peptides explained in Table 1 in stress-related conditions as well as in human health are well known, but their affinities as opioid peptides are still unclear. However, they are reported to exert opioid-like behaviors, but still, the mechanism of action is not well known. Therefore, further studies should be done to investigate their affinities as well as the mechanism of action as opioid peptides.

## 2. Classification of Opioid Peptides

They are divided into two categories as endogenous opioid peptides that are self-produced by the body and exogenous opioid peptides that are produced by different food sources such as plants and animals [25,26].

### 2.1. Endogenous Opioid Peptides

The endogenous opioid peptides are naturally produced in the mammalian system, which can operate as hormones (secreted by the gland and delivered to the target tissues) or neuromodulators (secreted by nerve cells and functioning in the central and peripheral nervous systems) [51]. In 1975 [16], the first endogenous ligand for opioid receptors was discovered and named as Enkephalins, and later, other endogenous peptides named Endorphins, Endomorphins, and Dynomorphins [10,52] were introduced. Opioid peptides that contain the conserved Tyr-Gly-Gly-Phe sequence at their *N* terminus are known as typical opioid peptides [51]. Therefore, for better understanding, Table 2A,B contains the amino acid sequences of endogenous opioid peptides, along with various binding affinities of endomorphin analogues. As it is well known, there are three opioid receptor types, µ-opioid receptor (MOR), δ-opioid receptor (DOR), and κ-opioid receptor and (KOR), which are responsible for the physiological and pharmacological effects of opioid peptides. Site-directed mutagenesis, receptor chimaera experiments, and NMR data have shown that MOR are the main opioid receptors, and μ-selectivity is primarily characterized by the second and third extracellular loops, as well as the intracellular carboxyl termini and extracellular amino termini of the MOR [53]. Apart from this, very significant features of the μ-selective agonists are the inclusion of Tyr^1^, Pro^2^, or d-Ala^2^ lipophilic residues at the third or fourth position and the amidation at the *C*-terminal [54]. So, with this theoretical basis, it would be possible to determine the conformational changes of the Endomorphins (EMs) belonging to the ligand–receptor binding and, eventually, to conjecture the detailed details of the MOR selectivity mechanism.

Enkephalins opioid peptides are present in the pituitary gland, brain, gastrointestinal tract, and kidney, and they are subdivided into two classes: Met-enkephalins and Leu-enkephalins. Another class of endogenous opioid peptides are endorphins, which are subdivided into four groups: α, β, γ, and σ. All are produced in the hypothalamus, pituitary gland, and in different parts of the nervous system and brain. Among all of them, β-endorphins are the most powerful one and play a crucial role as a neuromodulator [55]; they help to alleviate stress, body pain, and anxiety behaviors [55,56]. Endomorphins consist of types 1 and 2 endomorphins and dynorphins; both are located in the central nervous system and play significant roles in pain and stress-related conditions.

Using amino acid substitution, addition, deletion, cyclization, or the hybridization of two ligands, endogenous peptides have been modified into semisynthetic analogues to incorporate conformational constraints and make them more potent to be used as clinical analgesics [73]. Endomorphines have been transformed into analogues, which have improved protease stability by the addition of unnatural amino acids accompanied by cyclization [73,74,75]. Modifications of leu-enkephaline by the substitution, addition, and deletion of amino acids have resulted in a variety of agonists with improved δ receptor selectivity [51]. Extensive research has been done to synthesize analogues with the desired characteristics, which are not addressed in this review, but this information is available in different articles focusing only on therapeutics [20,73].

### 2.2. Food Derived Exogenous Opioid Peptides

These peptides are also known as exorphins having morphine-like activity, and they are derived exogenously, outside the body via different food sources. Dietary proteins are known as one of the essential sources of opioids because of their structural similarities to endogenous opioids [76]. Accumulating evidence suggests that opioid receptors may recognize peptides derived from the enzymatic hydrolysis of food proteins as they carry amino acid sequences with conserved tyrosine residue at the *N* terminus, and therefore, some of these opioid peptides are presented in Table 3 exhibit opioid activity along with physiological activity [77]. However, bovine α-casein peptides lacking tyrosine at the amino terminus Arg-Tyr-Leu-Gly-Tyr-Leu-Glu have also shown opioid activity [22]. To date, opioid peptides originating from animal proteins have been established as binding to μ receptors and those from plant proteins to δ receptors [78], except for soymorphins. Mostly exogenous opioid peptides are generated in the gastrointestinal tract and are absorbed in the bloodstream. They are also known to resist breakdown by intestinal enzymes such as proteases and can cross the blood–brain barrier to interact with opiate receptors [79]. There are various sources of these food-derived exogenous opioid peptides such as casein from human milk [19], β casein (buffalo milk) [25], and β-casomorphin (parmesan cheese [80], cheddar cheese [81]). Milk protein fermentation with lactic acid bacteria (LAB) is a desirable method for the production of functional foods enriched by bioactive peptides given its low cost and a good nutritional picture of fermented generated milk [82]. α-casein and β-casein are known as a good source of exogenous opioid peptides [76]. Milk was indicated to have opioid activity in 1979 [83], and morphine was isolated from milk as a drug at concentrations of 200 to 500 ng/L [84]. The opioid activity was attributed to the presence of β-casein [85] and f90-96 (Arg-Tyr-Leu-Gly-Tyr-Leu-Glu) and f90-95 (Arg-Tyr-Leu-Gly-Tyr-Leu) β-casein [86] peptides corresponding to f60-66 (Tyr-Pro-Phe-Pro-Gly-Pro-Ile, β-casomorphin-7). The opioid activity was also shown by the sequence corresponding to f91-96 (Tyr-Leu-Gly-Tyr-Leu-Glu) and f91-95 (Tyr-Leu-Gly-Tyr-Leu) amino acid residues, and Arg-Tyr-Leu-Gly-Tyr-Leu-Glu was the most potent [86]. β-casomorphin-7 from bovine β-casein was the first identified opioid peptide (Tyr-Pro-Phe-Pro-Gly-Pro-Ile) [87] and is known as the most potent opioid peptide in different βb-casomorphins (6, 5, and 4). As a result, β_h_-casomorphine-4,-5,-6 and-8 with Tyr-Pro-Phe-amino-termine were tested for opioid activity [57]. Based on the primary structure of human β-casein (β_h_-casein) and the sequence comparison with β_b_-casein, 10 residual shifted alignment relationships and 47% identity were established [57,88]. Moreover, β-casomorphins (BCM), which are produced by β-casein [89] (region 57–70), have shown potential effects on brain functions [89], calming, and sleep of infants [90] as well as in the modulation of behaviors such as anxiety [91]. Meanwhile, both β_h_-casomorphines and β_b_-casomorphines bind particularly to μ receptors, with the highest affinity for μ receptors and the lowest affinity for κ receptors [88].

Furthermore, there are various other food sources too that are reported as a source of exogenous opioid peptides such as barley (hordein peptide [79]), wheat (Gluten (gluten exorphins), a major wheat protein complex, Gliadin (gliadorphin), Glutenin (gluten morphin) peptides [55]), while gliadorphin-7 (Tyr-Pro-Gln-Pro-Gln-Pro-Phe) derived from α-gliadin has shown opioid activity [92]. In another study, whey protein consisting of β-lactoglobulin, immunoglobulins, α-lactalbumin, lactoperoxidase, lactoferrin, etc. was reported to exert opioid-like activity [57]. In contrast, soymorphins are known as specific ligands of the μ-opioid receptor, and many soymorphins were isolated from different sources showing opioid activities such as soymorphins 5 (Tyr-Pro-Phe-Val-Val), 6 (Tyr-Pro-Phe-Val-Val-Asn), and 7 (Tyr-Pro-Phe-Val-Val-Asn-Ala) [76]. Out of all three, soymorphin 5 (Tyr-Pro-Phe-Val-Val) has shown the highest opioid activity [93]. In another study, by the amidation or esterification, (by methyl group) of the peptides at carboxyl terminals such as valmuceptin (βh-casein 51–54 amide, Tyr-Pro-Phe-Val-NH_2_), morphiceptin (β-casein amide, Tyr-Pro-Phe-Pro-NH_2_), α-lactorphin (αh-lactalbumin 50–53 amide, Tyr-Gly-Leu-Phe-NH_2_), β-casorphin (β_h_-casein 41–44 amide, Try-Pro-Ser-Phe-NH2), β-casomorphin-4 and β-casomorphin-5 amides [94,95], several opioid peptide analogues have been identified. Lactoferroxin A (Tyr-Gly-Ser-Gly-Tyr-OCH_3_), B (Arg-Tyr-Tyr-Gly-Tyr-OCH_3_), and C (Lys-Tyr-Leu-Gly-Pro-Gln-Tyr-OCH_3_) are opioid antagonists derived from methyl-esterified human lactoferrin peptic digest [96]. The opioid activity of some of these peptides derived from food proteins is shown in Table 4.

Hydrolysate opioid activity can be assessed using one of many available assays. The naloxone-reversible inhibition of adenylate cyclase activity [97], naloxone-reversible inhibition of electrically induced contraction of isolated organ preparation, either mouse vas deferens and guinea-pig ileum [98], receptor binding assay or radio-receptor assay [99], are widely used assays for food opioid research. The main focus of the standard opioid activity determination tests in vitro was on µ and δ receptor interactions. These experiments are based on the inhibition of electrically evoked contractions of the mouse vas deferens (MVD) and the guinea pig ileum (GPI). The opioid effect in GPI preparations is primarily mediated by µ receptors, whereas the predominant MVD receptors are of the δ type [51]. Saturation and competition studies include receptor binding assays on tissue homogenates. The affinity of various compounds to opioid receptors is defined in saturation binding studies. Competition analyses can be performed subsequently or separately to validate these findings [51].

Exogenous opioid peptides have demonstrated promising effects in various investigations and the effects of administration of these opioid peptides at various doses in different animal models are presented in Table 5. Doses and results are difficult to compare, since various animal models and routes of administration have been used by different researchers. For example, Rubiscolin–6 improves memory consolidation [100], exerts orexigenic (oral administration) [101,102] and anxiolytic effects [103], and suppresses high-fat consumption [104]. β-casomorphine induces the release of somatostatin and insulin [105] and has been shown to prolong the gastrointestinal transit time [106] as well as modulate intestinal mucus secretion [107]. Apart from the central and peripheral nervous system effects by opioid receptors, β-casomorphin-7 also improved plasma insulin and superoxide dismutase and catalase activity in diabetic rats, thereby shielding them from hyperglycemia and free radical-mediated oxidative stress [108]. Gluten exorphin B5 enhanced the secretion of prolactin [109] and gluten exorphin C enhanced exploratory activity, improved learning, and decreased anxiety [110]; thus, studies show the positive effect of opioid peptides in human health.

The IC_50_ value is the concentration that would inhibit the electrically-evoked maximal contractions of the organ by 50%. As explained earlier, the opioid effect in GPI is primarily mediated by µ receptors, whereas in MVD receptors are of the δ type. So, the values in Table 4 represent the minimum concentration required for the activity with their selective receptors. For example, in soymorphins 5, 6, and 7, the IC_50_ value is lower in GPI assay as compared to values obtained by MVD assay, which shows there selectivity toward µ opioid receptors for activity.

Some current knowledge on the biological effects observed upon the intracerebroventricular, intraperitoneal, and oral administration of exogenous opioid peptides in the animal models are highlighted in Table 5. The fact that food-derived peptides can cross the small intestine and be present in blood and tissues has been appreciated for a long time [121,122,123]. In contrast, some pieces of evidence also suggests that [124] the degree of peptide absorption decreases with increasing chain length, so peptide length is also a point of consideration.

Although these studies (Table 5) provide useful information on the possible roles of the exogenous opioid peptides, still there are shortcomings in their research: for example, the exogenous peptides applied (similar to their endogenous counterparts) are unstable and are hydrolyzed into shorter forms, which typically have distinct activities after hydrolyzation from the parent peptide administered, leading to difficulties in the prediction of outcomes. So, technologies such as probe/radiolabeling will be effective here for the administration of parent peptides before hydrolysis inside the animal or human body. However, peptides can be secured against enzymatic cleavage by inserting a structure that induces a tail probe [125], by lactam bridge [126], by stacking or clipping peptide sequences [127], or by cycling [128].

For better understanding, deeper research with an explored signal cascade mechanism at the cellular and molecular level is needed to explore food-derived opioid peptides as therapeutic mediators, functional foods, or nutraceuticals for human health promotion.

## 3. Production of Opioid Peptides from Food Proteins

There are various emerging improvements in the methodologies for the analysis and the development of food-derived peptides. The main methods for their discovery can be classified as a traditional, in silico approach, or integrated approaches from the present literature.

### 3.1. The Traditional Approach

The traditional approach focuses on enzyme selection and production of the peptide through protein hydrolysis, purification of peptides, and peptide identification.

This is an extensively used method for recovering bioactive peptides from different food sources and comprises a collection of certain protein sources of interest. The proteins were usually digested with the help of food-grade enzymes (proteases, proteinases, or peptidases) to hydrolyze proteins into peptide fragments [129,130,131,132]. In addition to enzymes, various microorganisms may be used to ferment the proteins to enhance peptide breakdown [133,134,135]. The use of fermentation is possible because of the many proteolytic enzymes they possess for degrading proteins and to satisfy their nitrogen demands. Subsequently, the fermented peptides are extracted and purified according to their structural chemistry. The fractions undergo in vitro testing to evaluate their potential health effects. In an earlier study, β-casomorphin-7 was detected for the first time in the fermented sample [136]. In another study, gluten and exorphins were identified in a hydrolysate of pepsin and thermolysin [112] as well as in hydrolysate of pepsin–trypsin–chymotrypsin [113]. Gluten exorphins A5 with a weight around 0.747–2.192 mg/kg and C of 3.201–6.689 mg/kg have recently been found in bread and pasta following the simulation of in vitro GI digestion (using pepsin–trypsin–chymotrypsin) [137]. Similarly, casomorphin releases were analyzed in milk and its products after simulated GI digestion by different enzymes [138].

The hydrolysates with beneficial health effects are purified, and the active peptides were identified. Methods such as high-pressure liquid chromatography joined with mass spectrometry, reverse-phase liquid chromatography joined with mass spectrometry, and liquid chromatography-electrospray ionization along with quantitative time-of-flight tandem mass spectrometry have been used in studies to identify the bioactive peptides present in the sample [139,140,141]. This classical approach is effective in discovering new bioactive peptides from various protein substrates.

### 3.2. The In Silico Approach

**Figure 2:**
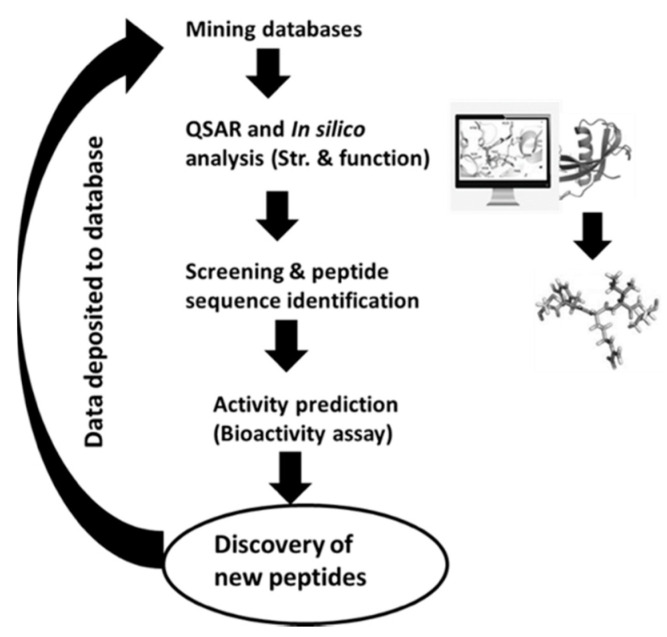
In silico approach to identify peptides from unknown sources of protein. QSAR: quantitative structure–activity relationship, Str: Structure.

The in silico methods comprise the use of information gathered from databases to find out the occurrence frequency of encrypted bioactive peptides in the primary food protein structure. The protein sequences can be accessible from databases for the analysis of different bioactive peptides (Table 6). As the existence of these peptides does not naturally mean the liberation of encrypted peptides, some bioinformatics software has been created that mimic proteolytic enzyme specificities to produce silico peptide profiles. Then, these proteases can be utilized for the hydrolysis of food protein, and their potential health effects were tested to build their efficacy. This method helps differentiate the identified peptides from unknown sources of protein (Figure 2). Most in silico platforms can predict the bioactive potential of the identified peptides, and the bioactivity can be confirmed through experiments in vitro and in vivo. As the in silico approach is rapid, cost-effective, and a greater number of options are available, various researchers are using this approach [142,143,144] to identify potent bioactive peptides. Proteolysis tools such as ExPASy Peptide Cutter (http:/web.expasy.org/peptide cutter), BIOPEP, and PoPS (http://pops.csse.monash.edu.au) are used to classify the specificities of different enzymes for releasing target peptide from food. By using this approach, sequences of cereal proteins (wheat, oat, barley, and rice) display high concentrations of peptides with dipeptidyl peptidase-inhibitory, anti-thrombotic, angiotensin-converting enzyme-inhibitory, antioxidant, hypotensive, and opioid activity [145]. In recent years, these computer-based databases have been used to predict the existence of bioactive peptides in food proteins [24,146,147,148]. Yet, more research is required to better understand this approach for the prediction of various food-based bioactive peptides.

### 3.3. Chemical Synthesis Approach

The key chemical approaches for peptide synthesis are solution-phase synthesis (SPS) and solid-phase peptide synthesis (SPPS). SPS is generally performed by coupling single amino acids in solution. Long peptide synthesis is feasible by synthesizing short fragments of the target peptides first and compressing them to produce long peptides [149]. This SPS approach is called the fragments condensation process. In the SPS method, it is possible to deprotect and purify intermediate products to achieve high purity of the target peptide [150,151]. SPS is economical and efficiently extensible, but the long reaction time remains a drawback. Meanwhile, the SPPS approach requires peptide synthesis using resin as a support for a growing peptide chain. An amino acid’s reactive side chain and α-amino group are first covered (mostly using the fluorenylmethoxycarbonyl protecting group (Fmoc) or tert-Butoxycarbonyl (Boc)) and the amino acids *C*-terminus is bound to the resin [152]. The *N*-terminal protecting group is normally removed (or cleaved) by using trifluoroacetic acid (Boc) or by 20% piperidine in *N*-*N*-dimethylformamide (Fmoc); then, the resin is washed before the introduction of subsequent amino acids. The peptide is separated off the resin after the required sequence is completed [149]. Presently, SPPS is widely used for therapeutic peptide synthesis because of its lower manufacturing costs and advancements in chromatographic equipment [149]. Long peptide or protein chains can also be synthesized by the chemical ligation approaches. The Native Chemical Ligation (NCL) is an efficient process for ligating peptides. A non-protected peptide segment containing an *N*-terminal cysteine is reacted to ligate peptide fragments with another unprotected peptide to form a thioester-linked intermediate, which is later reconstructed into a peptide bond. This process enables the formation of peptides of high molecular weight such as multivalent peptide-based non-symmetric dendrimer [153] and collagen-like polymers [154]. The advantages of this process are the high strength of the starting materials in NCL, the well-established chemical methods to manufacture peptide thioesters, and the high chemo-selective nature of the peptides. In addition, various researchers have used this approach such as Hartman et al. [155] for the treatment of pathological conditions of oxidation, Meisel et al. [156,157] for the oral administration of chemically synthesized peptides to mice in inflammation and atherosclerosis, González-García et al. [158] to transform bioactive peptide from waste to valuable product, Agyei et al. [159] to produce bioactive peptides to get large-scale recovery in pharmaceuticals, and Kim et al. [160] for the purification of bioactive peptides in food industries.

### 3.4. The Integrated Approach

**Figure 3:**
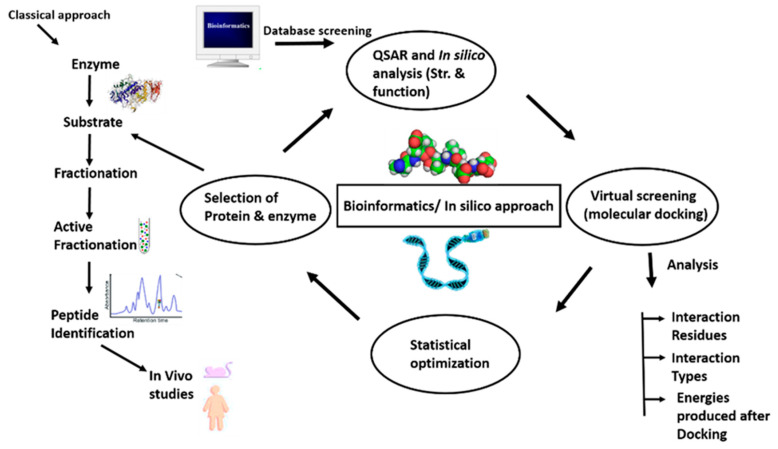
Representation of integrated approach (conventional/classical and bioinformatics) for the identification of food-derived bioactive peptides.

An integrated bioinformatics approach may be utilized in the detection of bioactive peptides because of limitations associated with the previous approaches. The strengths of both classical and bioinformatics approaches (Figure 3) can be combined to advance the analysis and need for peptides in health benefits and functional foods. The bioactive peptides determined in food proteins via the in silico method could be chemically synthesized, and this approach will lead to the detection of new peptides from new sources [161]. However, this is possible only if the whole sequence of a protein and the functional activities of the peptides are already known. Nonetheless, various important bioactive peptides of low concentration can further be missing if have not already been identified in the database. Therefore, alternative techniques such as peptide display methodologies were being used for the search of bioactive peptides at present.

### 3.5. Screening for Bioactive Peptides

After fermentation or enzymatic hydrolysis, it is usually very hard to identify particular bioactive peptides in a sample. Chemically synthesizing all of the peptides that could be found in a peptide digested for the screening purposes is costly and very laborious (if not impossible). Hence, many researchers use the bioassay fractionation approach, in which liquid chromatography separates protein digests into fractions. The fractions are analyzed, and potent fractions are further fractionated before mass spectrometry [162]. The peptides encrypted in the fractions are eventually recognized. While the approach has been successful and efficient, it typically omits the activity of lower concentrated fragments.

Using in silico platforms alone to predict bioactivity may be easy, but it may not be very accurate, as not all of the peptides predicted may be bioactive. Consequently, other researchers correlate their data collected from HPLC-MS along with many house databases that enhance the detection of less concentrated peptides [14]. However, several essential new low-concentration bioactive peptides can still be lost. In such a situation, the recombinant peptide libraries related to the coding sequence (peptide display) of these peptides can be used to identify dynamic bioactive peptides as an effective tool. Peptide display methodologies have been used as an effective research tool to track protein interactions at high throughput [163,164]. Phage display has been widely applied among most available molecular display techniques such as covalent antibody display, yeast and bacterial display, mRNA display, ribosome display, and CIS display. Phage display is a peptide selection approach that comprises the fusion of a peptide or protein with a protein coat of bacteriophage displayed on the virus surface [165]. The random peptide libraries displayed in a phage provide a functional approach to biopeptides, distinguishing peptides binding from those which are nonbinding peptides via affinity purification. The phage displayed random peptide library identification is an effective way of detecting peptides that can bind and control target molecular behaviors. This method has been practiced in identifying receptor-bound bioactive peptides [166], disease-specific antigen mimics [166,167], cell-specific peptides, non-protein-bound peptides [168], or organ-specific peptides [169], as well as in the designing of peptide-mediated drug delivery systems [165]. Consequently, analysis for bioactive peptides applying phage display technology is an advantageous approach that can be used in basic research. This way, the bioactive peptides achieved can be cloned and overexpressed to increase their quantity.

## 4. Mechanism of Action

**Figure 4:**
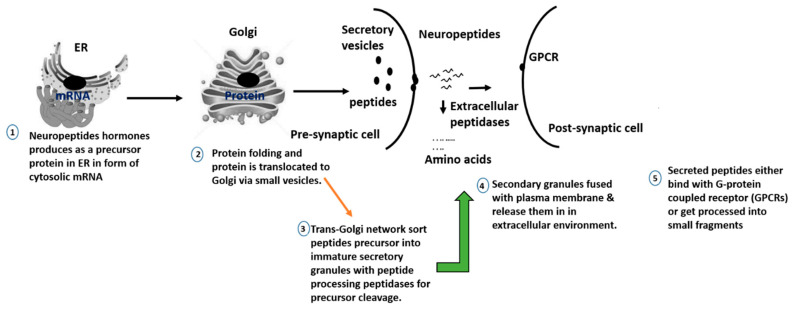
An overview of neuropeptide biosynthesis.

A receptor is a protein that binds with a chemical messenger and brings out an intracellular response. In the case of the opioid peptide, they either bind to a receptor or are converted into smaller peptides or amino acids (Figure 4). The receptors present are G-proteins coupled receptors (GPCRs); they are known as the largest gene family among all receptors, consisting of around 1000 different genes in human and other mammals [170].

**Figure 5:**
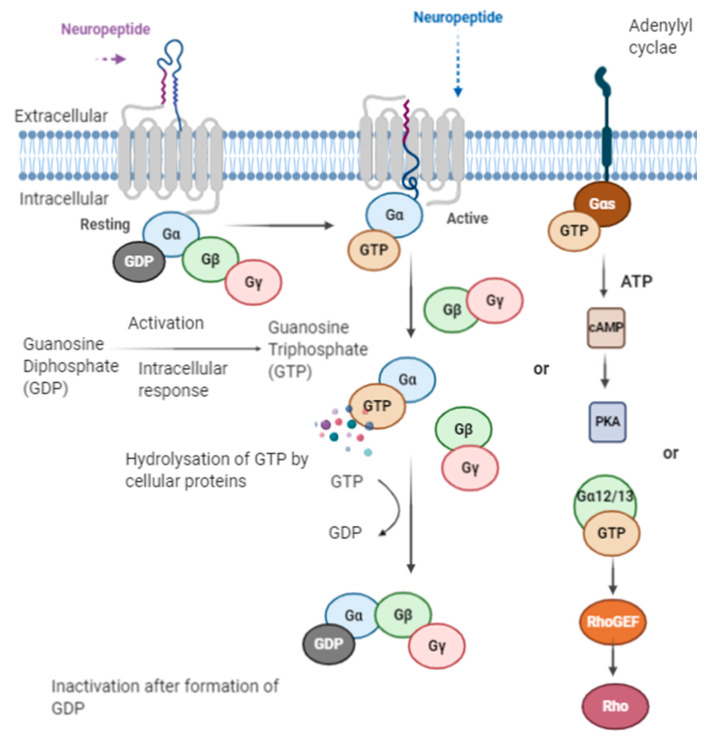
Mechanism of action.

When an opioid peptide binds to a receptor molecule, several intracellular changes of molecules occur along with other changes such as enzyme activation, the opening of ion channels, and the transcription of genes [170]. G-protein coupled receptors are also known as heptahelical receptors, as they consist of seven-transmembrane spanning receptors, and their signaling is conducted via G-proteins. Opioid peptides when functioning through GPCRs bind to the external surface of the receptor, which changes the conformation of the receptor proteins, leading to intracellular changes of the G proteins with the receptors [170]. The G-protein consists of 3 subunits α, β, and γ; when G-proteins are in an inactive state, the α-subunit binds with GDP (Guanosine diphosphate) along with other two subunits, β and γ, forming a G-protein complex. After receptor activation, the G-proteins attached GDP molecule is released and converted to a GTP (Guanosine triphosphate) molecule, and the GTP-α-subunit complex is dissociated from βγ subunits, which remain attached. The Gα-subunit is now able to modulate the activity of the effector molecules such as phospholipase or adenylyl cyclase [170]. Whereas it was also seen that in some cases, βγ subunits are also able to modulate some effector activities [170]. As soon as GTP bound with the α-subunit is hydrolyzed by cellular proteins, GTP hydrolyzed to GDP [170]. Thereafter, the free α-subunit again reassociates to form a heterotrimeric complex with βγ subunits, and this complex couples with the receptor for the next cycle of G-proteins upon activation (Figure 5). There are different subfamilies of G-proteins based on α-subunit Gi, Gs, Gq, and Go [76]. Everyone has multiple members who work via different pathways. The Gαs subunits activate adenylyl cyclase, whereas Gαo and Gαi subunits inhibit the adenylyl cyclase enzyme [76]. These activations lead to a series of reactions as adenylyl cyclase bring about cAMP(Cyclic adenosine monophosphate) formation from ATP, which activates protein kinase A. Earlier protein kinase A phosphorylates various intracellular substrates that result in biological modulations [76]. Moreover, Gαq subunits activate phospholipases that produce diacylglycerol and inositol 1,4,5-triphosphate [171]. Diacylglycerol activates protein kinase C for the phosphorylation of various molecules, whereas inositol 1,4,5-triphosphate activates receptors present on the endoplasmic reticulum for the opening of the Ca^2+^ channel [171]. Gα12/13 leads to the activation of guanine-nucleotide factors such as RhoGTPases for the exchange of GDP to proteins leading to biological changes [170].

These opioids peptide signaling systems are very complex as compared with some conventional neurotransmitters where no multiple receptor binding occurs and only a single ligand works with different receptors such as for acetylcholine [170]. Whereas most of them bind with multiple receptors but as an addition, they are also able to bind with receptors subtypes, creating more complexity in the signaling pathways. Yet, with the complexity of receptors and mechanism of action in these bioactive peptides, they still provide a promising and successful role in various clinical trials [170].

### Transport of Opioid Peptides in the Body

During digestion, digestive enzymes hydrolyze the food proteins to peptides and amino acids. Several factors influence the GI tract’s transport and absorption of peptides, including pKa, peptide size, and pH microclimate. Gastric emptying and the intestinal transit affect the location where the peptide is present along the GI tract and thus influence absorption. Peptides larger than di-tripeptides are found not easily absorbed in healthy people except in the conditions such as stress or disease when intestinal permeability is increased [172]. It has been seen that there is no absolute barrier to the intestinal mucosa and different peptides, including gluten exorphins A5 and A4, can cross the intestinal epithelium [57,137], whereas the mechanism of transfer is still not clear. In another study, it was observed that in a mammalian system, four different peptide transport systems PTS-1, PTS-2, PTS-3, and PTS-4 were involved that can transfer peptides, including food-derived peptides, from the peripheral circulation to the central nervous system through the blood–brain barrier [173]. In contrast, all four PTS-1 systems carry opioid peptides, including Tyr-MIF-1 met-enkephalin and leu-enkephalin [173]. Food-derived opioids absorbed in the gastrointestinal tract initially interact with receptors that are present on the enteric nervous system (ENS) and thus influence GI functions. The ENS is a network of nerve cells located in the GI tract wall, which control motility and secretion and regulate digestion, absorption, and immunomodulation [174]. In contrast, the glycosylation of peptides has also shown promising results in the transportation of peptides via the GLUT1 glucose carrier, along with the glycosylated analogues of dermorphin and met-encephalin [175]. However, due to peptidase activity, the half-life of opioid peptides in the blood is low. Endogenous opioids leu-enkephalin and dynorphin-A (1–13) have 6.7 min and 1 min half-lives [176,177]. In contrast, dermorphin exhibits a longer half-life than Enkephalins [178], and the half-life can be extended by binding these peptides to carrier proteins such as transferrin [179] or albumin [180]; some of the half-life stability data of opioid peptides are shown in Table 7. Therefore, in vivo half-life investigations of exogenous opioid peptides in blood need investigation for future research benefits.

## 5. Clinical and Animal Studies Related to Exogenous Opioid Peptides in Stress-Related Conditions

There are various preclinical and clinical studies available to support the role of several bioactive peptides in animals as well as in humans. Interestingly, as of now, opioid peptides are known as a potential target for the development of various new therapies related to stress disorders [184,185]. Here, we highlighted some pieces of evidence to support the role of food-derived peptides in stress-related conditions.

### Stress, Anxiety, and Depression

After the discovery of endogenous opioid peptides, experiments were performed to investigate exogenous peptides and their role in animal models. Studies related to exogenous opioid peptides have shown positive impacts on human health.

For instance, Lister et al. [91] had used various nociceptive models, and intracerebral (i.c.) or intracerebroventricular (i.c.v) routes were tested in which it was observed that various β-casomorphins (-3, -4, -5, -6, and -7) had shown anxiolytic effects. Limit et al. have found the effect of peptides isolated from soybean on brain functions [186]. Similarly, Bernet et al. explained the role of fish hydrolysates (Gabolysat PC60) on the levels of GABA and their sympathoadrenal activity leading anxiolytic effects in rats [187].

Additionally, rubiscolin-6 isolated from spinach RuBisCo was administered orally in mice and found to have an anxiolytic effect arbitrated by dopamine receptors [103] as well as a reduction of nociception in mice [188].

It was also seen that certain milk-based β-casomorphins interact with the receptors of opiates to affect the absorption of food in different conditions such as stress and anxiety [189]. Yin et al. has reported the protective role of β-casomorphins against oxidative stress [108]. In another, Chesnokova et al. [120] and Kaneko et al. [119] have shown the potential role of soymorphin-5 in the management of anxiety in mice.

On the other hand, soymorphin-5 demonstrated anxiolytic effects in mice, showing an improvement in the time mice remained in the open arms during an elevated plus-maze test [190]. In a human study, α-lactalbumin from full whey protein induced anxiolytic effects [191]. So, by going through different studies, we found that food-derived opioid peptides play a promising role in stress-related conditions as well as in human health.

## 6. Conclusions

In this review, we provided a detailed discussion on types of opioid peptides along with their sources, structures generated by enzyme hydrolysis of the food proteins, and their clinical pieces of evidence related to stress, anxiety, and depression. The opioid peptides have been discovered in the 1970s, and to our understanding, they hold great promise as valuable functional ingredients in healthy diets. In the modern generation, stress, anxiety, and depressive disorders are becoming a major issue, and food-derived opioid peptides showing anxiolytic and anti-stress effects can be a beneficial food substrate in maintaining a healthy population. The fermentation and enzymatic hydrolysis of food proteins are widely used in varying quantities and bioactivity to release these peptides. The amino acid profiles of various plants and animal food proteins suggest that these can become a great potential source for the production of bioactive peptides. Their effects are also dependent on their stability in blood, binding affinities, and half-lives as well as the capability of crossing the blood–brain barrier. The relative activity of these opioids depends on their affinity to the receptors, μ, δ, and κ. To date, opioid peptides from animal proteins tend to attach primarily to μ receptors, whereas those from plant proteins bind with δ receptors, except soymorphine. Most food opioid investigations were focused on widely used tissue preparations, mouse vas deferens, and guinea-pig ileum, which are unique to μ and δ receptors, respectively. It is not known whether the receptor of food-derived opioid peptides binds to the κ receptor. There is no proof in the literature of the use of rabbit vas deferens, which is known to be rich in κ opioid receptor, to confirm whether food opioids bind to this receptor [59].

On the other hand, various studies such as meta-analysis, animal model, and clinical examinations have shown the impact of these peptides on the nervous system (analgesia, antinociception, and improved memory), GI functions (increased intestinal transit time, increased appetite, and suppression of high fat intake), and increased β-oxidation and energy consumption, indicating the possibility of their use as nutraceuticals for pain relief, stress reduction, blood sugar, and obesity.

Still, to validate their roles, future consideration is needed to understand the stability of these peptides during digestion in animal and humans by in in vitro and in vivo studies as well as the health-related effects they generate across the gut–brain axis. Therefore, further research is required to understand and develop methods for the development of opioid peptides from food proteins in substantial quantities for pharmaceutical, beverage, and food use and system strategies to ensure their targeted distribution.

## Figures and Tables

**Table 1:** Opioid-like peptides and their roles.

Peptides	Functions	References
Colostrinin	Promotes acquisition of spatial learning in aged rats	[28]
Corticotropin-releasing factor (CRF)	Improves memory retention Enhance learning performance	[17,29]
Neuropeptide Y (NPY)	Neuroprotection as by control of feeding works against neurodegenerative diseases	[17,30]
Substance P (SP)	Improves functional recovery and increases the learning ability	[17,31]
Nociceptin/orphanin FQ (N/OFQ)	Impairs spatial learning in animal models. Facilitate memory	[32,33,34]
Angiotensin-vasopressin (AVP) and Oxytocin (OT)	Promote social memory and learning behaviors. Deficiency of AVP results in memory impairment	[35,36]
Cholecystokinin (CCK)	CCK peptides improve learning and memory performance in the patients Help in anxiety states Lack of CCK-A receptors cause impaired learning and memory functions. Play a role in conditioned fear stress and anxiety	[29,37,38]
Atrial natriuretic peptide (ANP), Brain-derived natriuretic peptide (BNP), *C*-type natriuretic peptide (CNP)	Promote action on memory consolidation	[39,40]
Pituitary adenylate cyclase-activating polypeptide (PACAP)	Promote learning (consolidation and retrieval)	[41]
Galanin	Impairs the learning and memory performances overexpression impairs cognition	[42,43,44]
Bombesin/gastrin-releasing peptide (BN/GRP) and Neuromedin (NM)	Improve memory performance	[45,46]
Hippocampal cholinergic neurostimulation Peptide (HCNP)	Abnormal accumulation and expression associated with memory and learning disorders	[47]
Calcitonin-gene related peptide (CGRP), Substance P(SP) and Neuropeptide Y (NPY)	Enhance memory retention. SP improves functional recovery and increases learning ability. NPY enhances memory	[30]
Insulin	Improves short-term memory	[48]
Orexin-A	Inhibits long-term potentiation (LTP) and retards spatial learning	[49,50]

**Table 2:** (**A**) Endogenous opioid peptides. (**B**) Binding affinities of various Endomorphin analogues.

(**A**)				
**Opioid Peptide**	**Amino-Acid Sequence**		**Protein Precursor**	**References**
endomorphin-1	Tyr-Pro-Trp-Phe-NH_2_		pro-endomorphin	[57,58]
endomorphin-2	Tyr-Pro-Phe-Phe-NH_2_		pro-endomorphin	[57,58]
met-enkephalin	Tyr-Gly-Gly-Phe-Met		pro-enkephalin	[16],
leu-enkephalin	Tyr-Gly-Gly-Phe-Leu		pro-enkephalin	[16,59]
β-endorphin	Tyr-Gly-Gly-Phe-Met-Thr-Ser-Glu-Lys-Ser-Gln-ThrPro-Leu-Val-Thr-Leu-Phe-Lys-Asn-Ala-Ile-Ile-LysAsn-Ala-Tyr-Lys-Lys-Gly-Glu		pro-opiomelanocortin	[60,61]
dynorphin A	Tyr-Gly-Gly-Phe-Leu-Arg-Arg-Ile-Arg-Pro-Lys-LeuLys-Trp-Asp-Asn-Gln		pro-dynorphin	[62,63]
dynorphin B	Phe-Gly-Gly-Phe-Thr-Gly-Ala-Arg-Lys-Ser-Ala-ArgLys-Leu-Ala-Asn-Gln		pronociceptin	[62,63]
(**B**)				
**Sequence**	**IC_50_ (nM)**		**Ratio of IC_50_ Ratio δ/µ**	**References**
	**Affity (µ-Receptor)**	**Affity (δ-Receptor)**		
Endomorphins Modified at First Amino Acid Position				
d-Tyr^1^-Pro-Phe-Phe-NH_2_	32.1 ± 1.5	4121 ± 1492	128	[64]
Dmt^1^-Pro-Trp-Phe-NH_2_	0.014 ± 0.003	12.0 ± 4.05	857	[65]
Mmt^1^-Pro-Phe-Phe-NH_2_	0.132 ± 0.008	528.6 ± 47	4005	[65]
Emt^1^-Pro-Phe-Phe NH_2_	0.063 ± 0.006	55.7 ± 6.2	884	[65]
Dit^1^-Pro-Phe-Phe-NH_2_	2.29 ± 0.37	105 ± 16	46	[65]
Det^1^-Pro-Phe-Phe-NH_2_	0.084 ± 0.006	69.7 ± 5.3	830	[65]
Tmt^1^-Pro-Phe-Phe-NH_2_	1.111 ± 0.002	593.5 ± 80	5347	[65]
Endomorphins Modified at Second Amino Acid Position				
Tyr-d-Pro^2^-Phe-Phe-NH_2_	512.4 ± 29	30,641 ± 419	60	[64]
Tyr-Aze^2^-Trp-Phe-NH_2_	2.3 ± 0.23	3500 ± 360	1500	[66]
Tyr-δAla^2^-Phe-Phe-NH_2_	34 ± 6.3	710 ± 130	21	[66]
Tyr-3Aze^2^-Phe-Phe-NH_2_	210 ± 51	6900 ± 1200	32	[66]
Tyr-Aze^2^-Phe-Phe-NH_2_	5.6 ± 1.2	5100 ± 600	920	[66]
Endomorphins Modified at Third Amino Acid Position				
Tyr-Pro-d-Phe^3^-Phe-NH_2_	203.2 ± 83	4230 ± 344	21	[64]
Tyr-Pro-Phe^3^-(*p*-NH_2_)-Phe-NH_2_	185 ± 36	>10,000	>1.9	[67]
TyrProPhe^3^(*p*-NHCOCH_2_Br)-PheNH_2_	7210 ± 820	>10,000	>1.4	[67]
Tyr-Pro-(*2S,3R*)-βMePhe^3^-Phe-NH_2_	106 ± 9	>10,000	>10	[68]
Tyr-Pro-(*2S,3S*)-βMePhe^3^-Phe-NH_2_	45.3 ± 4.1	179 ± 15	4	[68]
Tyr-Pro-(*2R,3S*)-βMePhe^3^-Phe-NH_2_	4910 ± 328	>10,000	>2	[68]
Tyr-Pro-(*2R,3R*)-βMePhe^3^-Phe-NH_2_	7090 ± 131	6760 ± 865	1	[68]
Tyr-Pro-(F_5_)-Phe^3^-Phe-NH_2_	11.7 ± 0.503	11,700 ± 1010	1000	[69]
Endomorphins Modified at C-TERMINAL Position				
Tyr-Pro-Phe-d-Phe^4^-NH_2_	45.9 ± 8.6	8159 ± 1569	177	[64]
Tyr-Pro-Phe-(*p*-NH_2_)-Phe^4^-NH_2_	36.7 ± 2.2	>10,000	>270	[67]
TyrProPhePhe^4^(*p*-NHCOCH_2_Br)-NH_2_	158 ± 23	1940 ± 310	12	[67]
Tyr-Pro-Phe-(*p*-NCS)-Phe^4^-NH_2_	345 ± 128	>10,000	>29	[67]
Tyr-Pro-Trp-Dmp^4^-NH_2_	13.2 ± 1.9	7624 ± 2571	578	[70]
Tyr-Pro-Trp-d-Dmp^4^-NH_2_	106 ± 20	1765 ± 834	17	[70]
Tyr-Pro-Phe-Phe^4^-NH-(CH_2_)_5_-CODap(6DMN)-NH_2_	244.5 ± 14	5939 ± 1396	24	[71]
Tyr-Pro-Phe-d-Val^4^-NH-Bn	4.97 ± 1.24	3358 ± 414	676	[72]
Tyr-Pro-Trp-d-Val^4^-NH-Bn	2.32 ± 0.15	3287 ± 456	1417	[72]

2′,6′-dimethyltyrosine (Dmt), 2′-monomethyltyrosine (Mmt), 2′,3′,6′-trimethyltyrosine (Tmt), 2′-ethyl-6′-methyltyrosine (Emt), 2′,6′-diethyltyrosine (Det), 2′6′-dimethylphenylalanine (Dmp), 6-*N*,*N*-(dimethylamino)-2,3-naphthalimide (6DMN) and 2′,6′-diisopropyltyrosine (Dit).

**Table 3:** Exogenous food-derived opioid peptides.

Source	Sequences	Peptide Name	References
Bovine milk β-casein	Tyr-Pro-Phe-Pro	β_b_-casomorphin-4	[87]
	Tyr-Pro-Phe-Pro-Gly	β_b_-casomorphin-5	
	Tyr-Pro-Phe-Pro-Gly-Pro	β_b_-casomorphin-6	
	Tyr-Pro-Phe-Pro-Gly-Pro-Ile	β_b_-casomorphin-7	
	Tyr-Pro-Val-Glu-Pro-Phe	Neocasomorphin-6	[111]
Bovine milk α-lactalbumin	Tyr-Gly-Leu-Phe-NH_2_	α_b_-lactorphin	[22]
Human milk β-casein	Tyr-Pro-Phe-Val	β_h_-casomorphin-4	[22]
	Tyr-Pro-Phe-Val-Glu	β_h_-casomorphin-5	[57]
	Tyr-Pro-Phe-Val-Glu-Pro-Ile	β_h_-casomorphin-7	[88]
	Tyr-Pro-Phe-Val-Glu-Pro-Ile-pro	β_h_-casomorphin-8	[22,88]
Human milk lactalbumin	Tyr-Gly-Leu-Phe-NH_2_	α_h_-lactorphin	[22,94]
Bovine/bovine milk lactoferrin	Tyr-Leu-Gly-Ser-Gly-Tyr-OCH_3_	lactoferrsoxin A	[96]
	Arg-Tyr-Tyr-Gly-Tyr-OCH_3_	lactoferrsoxin B	
	Lys-Tyr-Leu-Gly-Pro-Gln-Tyr-OCH_3_	lactoferrsoxin C	
Soy β-conglycinin	Tyr-Pro-Phe-Val-Val	Soymorphin-5	[93]
	Tyr-Pro-Phe-Val-Val-Asn	Soymorphin-6	
	Tyr-Pro-Phe-Val-Val-Asn-Ala	Soymorphin-7	
Wheat HMW glutenin	Gly-Tyr-Tyr-Pro	gluten exorphin A4	[78,112]
	Gly-Tyr-Tyr-Pro-Thr	gluten exorphin A5	
	Tyr-Gly-Gly-Trp	gluten exorphin B4	
	Tyr-Gly-Gly-Trp-Leu	gluten exorphin B5	
	Tyr-Pro-Ile-Ser-Leu	gluten exorphin C	[78,113]
Spinach RuBisCo	Tyr-Pro-Leu-Asp-Leu	rubiscolin-5	[93,114]
	Tyr-Pro-Leu-Asp-Leu-Phe	rubiscolin-6	

**Table 4:** The opioid activity of exogenous peptides (IC50 in µM).

Opioid Peptide	Opioid Activity (IC50 in µM)		µ/δ Ratio	Reference
	Mouse (vas Deferens) (δ)	Guinea-Pig (ileum) (µ)		
rubiscolin-5	51	1110	21.8	[114]
rubiscolin-6	24.4	748	30.7	[114]
β_b_-casomorphin-4	84	22	0.26	[87]
β_b_-casomorphin-5	40	6.5	0.16	[87]
β_b_-casomorphin-6	>150	27.4	<0.18	[87]
β_b_-casomorphin-7	>200	57	<0.29	[87]
β_h_-casomorphin-4	750	19	0.025	[94]
β_h_-casomorphin-5	ND	14	ND	[94]
β_h_-casomorphin-6	350	25	0.071	[94]
β_h_-casomorphin-8	540	25	0.047	[94]
gluten-exorphin A4	70	>1000	ND	[112]
gluten exorphin A5	60	1000	60.7	[112]
gluten exorphin B4	3.4	1.5	0.44	[112]
gluten exorphin B5	0.017	0.05	2.9	[112]
gluten exorphin C	30	110	3.7	[113]
soymorphin-5	50	6	0.12	[93]
soymorphin-6	32	9.2	0.287	[93]
soymorphin-7	50	13	0.26	[93]
Human milk lactalbumin (α-lactorphin)	>1000	50	ND	[94]
Bovine milk lactoferrin (lactoferrsoxin)	4.38	5.68	0.77	[96]

ND—not determined, IC_50_ is the 50% inhibitory concentration.

**Table 5:** Trials of exogenous opioid peptides in animal models.

Opioid Peptide	Animal Model	Dosage	Administration Route	Effect	Time Duration	Reference
rubiscolin-5	Mice	3 nM/mouse	i.c.v	antinociception	Effects observed up to 30 min post-injection	[114]
rubiscolin-6	Mice	1 nM/mouse	i.c.v			
rubiscolin–6	Mice	100 mg/kg 3 nM/mouse	Oral i.c.v	enhancement in memory consolidation	Effects observed up to 24 h post-injection	[100]
β-casomorphin- 4,-5,-6,-7	Rat	60–2000 nM	i.c.v	analgesic, naloxone reversible	Effects observed up to 30–40 min post-injection	[115]
β-casomorphin-5	Mice Rat	1mg/kg 166 nM	i.p i.v	improvement in learning and memory, analgestic	Effects observed up to 30-min post-injection Significant analgesia l0-min after injection up to 60 min post-injection	[116,117]
β-casomorphin-7	Rat	0.1–20 nM	i.c.v	food-intake stimulation	Effects observed up to 6 h post-injection	[118]
gluten exorphin C	Mice	5mg/kg	i.p	improvement in learning and behavior, decreased anxiety	Effects observed up to 15–20 min post-injection	[110]
Gluten-exorphin B5	Rat	3 mg/kg	i.v	stimulated prolactin secretion	Effects observed after 20 min post-injection	[109]
soymorphin-5, 6, and 7	Mice	10–30 mg/kg or 3 mg/kg	Oral i.p	anxiolytic effect	Oral—Effects observed up to 20–25 min post-injection i.p—Effects observed <30min of post-injection	[93]
soymorphin-5 and 7	Mice	30 mg/kg or 48 µ mol/kg	oral	reduced food intake and showed anorexigenic activity	Effects observed after 2 h of oral administration	[119]
soymorphin-5 amide	Rat	5 mg/kg	i.p	decreased anxiety	Effects observed after 30 min of administration	[120]

i.c.v—intracerebroventricular; i.p—intraperitoneal; i.v—intravenous; nM—nano mol.

**Table 6:** In silico databases and online tools for analysis of bioactive peptides.

Databases Name	Address	Role
NeuroPIpred	https://webs.iiitd.edu.in/raghava/neuropipred	Neuropeptide database
NeuroPP	http://i.uestc.edu.cn/neuropeptide/neuropp/home.html	Neuropeptide database
BIOPEP (Bioactivity) (digestion) (Protein) (toxicity)	http://www.uwm.edu.pl/biochemia/index.php/en/biopep	Prediction for precursors of bioactive peptides,
ToxinPred (Toxicity)	http://crdd.osdd.net/raghava//toxinpred/	Prediction of toxicity of peptides
I-TASSER (Protein Structure)	https://zhanglab.ccmb.med.umich.edu/I-TASSER/	Structure and function prediction
NCBI (Protein Database)	https://www.ncbi.nlm.nih.gov/	Protein sequences information
AlgPred (Toxicity)	http://crdd.osdd.net/raghava//algpred/	Prediction of toxicity of peptides
ProtParam (phytochemical)	http://web.expasy.org/protparam/	Compute GRAVY (grand average of hydropathicity)
UniProtKB (Protein database)	http://www.uniprot.org/	Structure and sequences information
APD (Peptide database)	http://aps.unmc.edu/AP/main.html	Bioactive peptide prediction
AntiBP2 (Bioactivity prediction)	http://crdd.osdd.net/raghava//antibp2/	Antibacterial peptide prediction
PEPstrMOD (Peptide database)	http://osddlinux.osdd.net/raghava/pepstrmod	Prediction of tertiary structures

**Table 7:** Stability half-life of opioid peptides.

Sequence	Half-Life (Mouse Brain) [min]	Reference
[d-Ala^2^, *p*-Cl-Phe^4^]EM-1	>300	[181]
[Dmt^1^,Nip^2^]EM-1	30.9 ± 3.29	[182]
[(*2S,3S*)β-MePhe^4^]EM-2	35.8 ± 1.8	[68]
[(*1S,2R*)ACHC^2^]EM-1	>12 h	[183]
Guanidino-[d-Pro^2^Gly^3^, *p*-Cl-Phe^5^]EM-1	187.3 ± 24	[181]
[(*1S,2R*)ACPC^2^]EM-2	>12 h	[183]
[Dmt^1^,Nip^2^]EM-2	10.7 ± 0.3	[182]
Guanidino-[d-Pro^2^-Gly^3^]EM-1	111.8 ± 19.2	[181]
Guanidino-[Sar^2^]EM-1	43.9 ± 2.4	[181]

EM-1—endomorphin 1, EM-2—endomorphin 2, cis-/trans-2-aminocyclopentanecarboxylic acid (ACPC), cis-/trans-2-aminocyclohexanecarboxylic acid (ACHC), piperidine-3-carboxylic acid (Nip), 2′6′-dimethyltyrosine (Dmt).

## References

[1] World Health Organization World Mental Health Day, 10 October 2012. http://www.who.int/mental_health/management.

[2] Mills K.T., Stefanescu A., He J. (2020). The global epidemiology of hypertension. Nat. Rev. Nephrol..

[3] Szelenberger W., Soldatos C. (2005). Sleep disorders in psychiatric practice. World Psychiatry.

[4] Substance Abuse and Mental Health Services Administration (2017). Mental Health Services Administration. Key Substance Use and Mental Health Indicators in the United States: Results from the 2016 National Survey on Drug Use and Health (HHS Publication No. SMA 17-5044, NSDUH Series H-52).

[5] Ned H., Kalin M.D. (2020). The Critical Relationship Between Anxiety and Depression. Am. J. Psychiatry.

[6] Kessler R.C., Sampson N.A., Berglund P., Gruber M., Al-Hamzawi A., Andrade L., Bunting B., Demyttenaere K., Florescu S., De Girolamo G. (2015). Anxious and non-anxious major depressive disorder in the World Health Organization World Mental Health Surveys. Epidemiol. Psychiatr. Sci..

[7] Gee D.G., Kribakaran S. (2020). Developmental Differences in Neural Responding to Threat and Safety: Implications for Treating Youths with Anxiety. Am. Psychiatr. Assoc..

[8] Poppitt S.D. (2020). Milk proteins and human health. Milk Proteins.

[9] González S. (2020). Dietary Bioactive Compounds and Human Health and Disease. Nutrients.

[10] Daliri E.B.-M., Lee B.H., Oh D.H. (2018). Current trends and perspectives of bioactive peptides. Crit. Rev. Food Sci. Nutr..

[11] Daliri E.B.-M., Oh D.H., Lee B.H. (2017). Bioactive peptides. Foods.

[12] Daliri E.B.-M., Lee B.H., Park M.H., Kim J.-H., Oh D.-H. (2018). Novel angiotensin I-converting enzyme inhibitory peptides from soybean protein isolates fermented by Pediococcus pentosaceus SDL1409. LWT.

[13] Daliri E.B.-M., Ofosu F.K., Chelliah R., Park M.H., Kim J.-H., Oh D.-H. (2019). Development of a soy protein hydrolysate with an antihypertensive effect. Int. J. Mol. Sci..

[14] Daliri E.B.-M., Lee B.H., Park B.-J., Kim S.-H., Oh D.-H. (2018). Antihypertensive peptides from whey proteins fermented by lactic acid bacteria. Food Sci. Biotechnol..

[15] Martínez-Villaluenga C., Hernández-Ledesma B. (2020). Peptides for Health Benefits 2019. Int. J. Mol. Sci..

[16] Hughes J., Smith T., Kosterlitz H., Fothergill L.A., Morgan B., Morris H. (1975). Identification of two related pentapeptides from the brain with potent opiate agonist activity. Nature.

[17] Gulpinar M.A., Yegen B.C. (2004). The Physiology of Learning and Memory: Role of Peptides and Stress. Curr. Protein Pept. Sci..

[18] Wang Y., Van Bockstaele E.J., Liu-Chen L.-Y. (2008). In vivo trafficking of endogenous opioid receptors. Life Sci..

[19] Rutherfurd-Markwick K.J. (2012). Food proteins as a source of bioactive peptides with diverse functions. Br. J. Nutr..

[20] Bodnar R.J. (2013). Endogenous opiates and behavior: 2012. Peptides.

[21] Bagley E.E., Ingram S.L. (2020). Endogenous opioid peptides in the descending pain modulatory circuit. Neuropharmacology.

[22] Teschemacher H., Koch G., Brantl V. (1997). Milk protein-derived opioid receptor ligands. Pept. Sci..

[23] Meisel H., Fitzgerald R.J. (2000). Opioid peptides encrypted in intact milk protein sequences. Br. J. Nutr..

[24] Carrasco-Castilla J., Hernández-Álvarez A.J., Jiménez-Martínez C., Gutiérrez-López G.F., Dávila-Ortiz G. (2012). Use of proteomics and peptidomics methods in food bioactive peptide science and engineering. Food Eng. Rev..

[25] Trivedi M.S., Shah J.S., Al-Mughairy S., Hodgson N.W., Simms B., Trooskens G.A., Van Criekinge W., Deth R.C. (2014). Food-derived opioid peptides inhibit cysteine uptake with redox and epigenetic consequences. J. Nutr. Biochem..

[26] Bhat Z., Kumar S., Bhat H.F. (2015). Bioactive peptides of animal origin: A review. J. Food Sci. Technol..

[27] Koyama M., Hattori S., Amano Y., Watanabe M., Nakamura K. (2014). Blood pressure-lowering peptides from neo-fermented buckwheat sprouts: A new approach to estimating ACE-inhibitory activity. PLoS ONE.

[28] Popik P., Bobula B., Janusz M., Lisowski J., Vetulani J. (1999). Colostrinin, a polypeptide isolated from early milk, facilitates learning and memory in rats. Pharmacol. Biochem. Behav..

[29] Gülpınar M.A., Özbeyli D., Arbak S., Yeğen B.Ç. (2004). Anti-inflammatory effect of acute stress on experimental colitis is mediated by cholecystokinin-B receptors. Life Sci..

[30] Bracci-Laudiero L., Aloe L., Lundeberg T., Theodorsson E., Stenfors C. (1999). Altered levels of neuropeptides characterize the brain of lupus prone mice. Neurosci. Lett..

[31] Sprick U., Hasenöhrl R., Krauth J., Klapdor K., Huston J. (1996). Effects of chronic substance P treatment and intracranial fetal grafts on learning after hippocampal kainic acid lesions. Peptides.

[32] Sandin J., Georgieva J., Schött P.A., Ögren S.O., Terenius L. (1997). Nociceptin/orphanin FQ microinjected into hippocampus impairs spatial learning in rats. Eur. J. Neurosci..

[33] Manabe T., Noda Y., Mamiya T., Katagiri H., Houtani T., Nishi M., Noda T., Takahashi T., Sugimoto T., Nabeshima T. (1998). Facilitation of long-term potentiation and memory in mice lacking nociceptin receptors. Nature.

[34] Hiramatsu M., Inoue K. (2000). Improvement by low doses of nociceptin on scopolamine-induced impairment of learning and/or memory. Eur. J. Pharmacol..

[35] Lynch G., Larson J., Staubli U., Granger R. (1991). Variants of Synaptic Potentiation and Different Types of Memory Operations in Hippocampus and Related Structures.

[36] Chen Q., Patel R., Sales A., Oji G., Kim J., Monreal A., Brinton R. (2000). Vasopressin-induced neurotrophism in cultured neurons of the cerebral cortex: Dependency on calcium signaling and protein kinase C activity. Neuroscience.

[37] Crawley J.N., Corwin R.L. (1994). Biological actions of cholecystokinin. Peptides.

[38] Dauge V., Pophillat M., Crete D., Melik-Parsadaniantz S., Roques B. (2003). Involvement of brain endogenous cholecystokinin in stress-induced impairment of spatial recognition memory. Neuroscience.

[39] Telegdy G., Adamik A., Glover V. (2000). The action of isatin (2, 3-dioxoindole) an endogenous indole on brain natriuretic and C-type natriuretic peptide-induced facilitation of memory consolidation in passive-avoidance learning in rats. Brain Res. Bull..

[40] Telegdy G., Kokavszky K., Nyerges A. (1999). Action of C-type natriuretic peptide (CNP) on passive avoidance learning in rats: Involvement of transmitters. Eur. J. Neurosci..

[41] Telegdy G., Kokavszky K. (2000). The action of pituitary adenylate cyclase activating polypeptide (PACAP) on passive avoidance learning. The role of transmitters. Brain Res..

[42] McDONALD M.P., Gleason T.C., Robinson J.K., Crawley J.N. (1998). Galanin inhibits performance on rodent memory tasks. Ann. N. Y. Acad. Sci..

[43] Counts S.E., Perez S.E., Kahl U., Bartfai T., Bowser R.P., Deecher D.C., Mash D.C., Crawley J.N., Mufson E.J. (2001). Galanin: Neurobiologic mechanisms and therapeutic potential for Alzheimer’s disease. CNS Drug Rev..

[44] Wrenn C., Marriott L., Kinney J., Holmes A., Wenk G., Crawley J. (2002). Galanin peptide levels in hippocampus and cortex of galanin-overexpressing transgenic mice evaluated for cognitive performance. Neuropeptides.

[45] Santo-Yamada Y., Yamada K., Wada E., Goto Y.-I., Wada K. (2003). Blockade of bombesin-like peptide receptors impairs inhibitory avoidance learning in mice. Neurosci. Lett..

[46] Yamada K., Santo-Yamada Y., Wada K. (2003). Stress-induced impairment of inhibitory avoidance learning in female neuromedin B receptor-deficient mice. Physiol. Behav..

[47] Ojika K., Mitake S., Tohdoh N., Appel S.H., Otsuka Y., Katada E., Matsukawa N. (2000). Hippocampal cholinergic neurostimulating peptides (HCNP). Prog. Neurobiol..

[48] Gasparini L., Netzer W.J., Greengard P., Xu H. (2002). Does insulin dysfunction play a role in Alzheimer’s disease?. Trends Pharmacol. Sci..

[49] Moriguchi T., Sakurai T., Nambu T., Yanagisawa M., Goto K. (1999). Neurons containing orexin in the lateral hypothalamic area of the adult rat brain are activated by insulin-induced acute hypoglycemia. Neurosci. Lett..

[50] Aou S., Li X.-L., Li A.-J., Oomura Y., Shiraishi T., Sasaki K., Imamura T., Wayner M. (2003). Orexin-A (hypocretin-1) impairs Morris water maze performance and CA1-Schaffer collateral long-term potentiation in rats. Neuroscience.

[51] Janecka A., Fichna J., Janecki T. (2004). Opioid receptors and their ligands. Curr. Top. Med. Chem..

[52] Koneru A., Satyanarayana S., Rizwan S. (2009). Endogenous opioids: Their physiological role and receptors. Glob. J. Pharm..

[53] Law P.-Y., Wong Y.H., Loh H.H. (1999). Mutational analysis of the structure and function of opioid receptors. Pept. Sci..

[54] Yang Y.-R., Chiu T.-H., Chen C.-L. (1999). Structure–activity relationships of naturally occurring and synthetic opioid tetrapeptides acting on locus coeruleus neurons. Eur. J. Pharmacol..

[55] Kaur J., Kumar V., Sharma K., Kaur S., Gat Y., Goyal A., Tanwar B. (2020). Opioid Peptides: An Overview of Functional Significance. Int. J. Pept. Res. Ther..

[56] Froehlich J.C. (1997). Opioid peptides. Alcohol Health Res. World.

[57] Garg S., Nurgali K., Kumar Mishra V. (2016). Food proteins as source of opioid peptides-a review. Curr. Med. Chem..

[58] Hackler L., Zadina J.E., Ge L.-J., Kastin A.J. (1997). Isolation of relatively large amounts of endomorphin-1 and endomorphin-2 from human brain cortex. Peptides.

[59] Aldrich J.V., Kulkarni S.S., Senadheera S.N., Ross N.C., Reilley K.J., Eans S.O., Ganno M.L., Murray T.F., McLaughlin J.P. (2011). Unexpected Opioid Activity Profiles of Analogues of the Novel Peptide Kappa Opioid Receptor Ligand CJ-15,208. ChemMedChem.

[60] Goldstein A. (1976). Opioid peptides (endorphins) in pituitary and brain. Science.

[61] Li C.H., Chung D. (1976). Isolation and structure of an untriakontapeptide with opiate activity from camel pituitary glands. Proc. Natl. Acad. Sci. USA.

[62] Goldstein A., Fischli W., Lowney L.I., Hunkapiller M., Hood L. (1981). Porcine pituitary dynorphin: Complete amino acid sequence of the biologically active heptadecapeptide. Proc. Natl. Acad. Sci. USA.

[63] Chavkin C., James I.F., Goldstein A. (1982). Dynorphin is a specific endogenous ligand of the kappa opioid receptor. Science.

[64] Okada Y., Fukumizu A., Takahashi M., Shimizu Y., Tsuda Y., Yokoi T., Bryant S.D., Lazarus L.H. (2000). Synthesis of stereoisomeric analogues of endomorphin-2, H-Tyr-Pro-Phe-Phe-NH2, and examination of their opioid receptor binding activities and solution conformation. Biochem. Biophys. Res. Commun..

[65] Li T., Fujita Y., Tsuda Y., Miyazaki A., Ambo A., Sasaki Y., Jinsmaa Y., Bryant S.D., Lazarus L.H., Okada Y. (2005). Development of Potent μ-Opioid Receptor Ligands Using Unique Tyrosine Analogues of Endomorphin-2. J. Med. Chem..

[66] Torino D., Mollica A., Pinnen F., Lucente G., Feliciani F., Davis P., Lai J., Ma S.-W., Porreca F., Hruby V.J. (2009). Synthesis and evaluation of new endomorphin analogues modified at the Pro2 residue. Bioorgan. Med. Chem. Lett..

[67] Choi H., Murray T.F., Aldrich J.V. (2003). Synthesis and evaluation of potential affinity labels derived from endomorphin-2. J. Pept. Res..

[68] Tömböly C., Kövér K.E., Péter A., Tourwé D., Biyashev D., Benyhe S., Borsodi A., Al-Khrasani M., Rónai A.Z., Tóth G. (2004). Structure−Activity Study on the Phe Side Chain Arrangement of Endomorphins Using Conformationally Constrained Analogues. J. Med. Chem..

[69] Honda T., Shirasu N., Isozaki K., Kawano M., Shigehiro D., Chuman Y., Fujita T., Nose T., Shimohigashi Y. (2007). Differential receptor binding characteristics of consecutive phenylalanines in μ-opioid specific peptide ligand endomorphin-2. Bioorgan. Med. Chem..

[70] Sasaki Y., Sasaki A., Niizuma H., Goto H., Ambo A. (2003). Endomorphin 2 analogues containing Dmp residue as an aromatic amino acid surrogate with high μ-opioid receptor affinity and selectivity. Bioorgan. Med. Chem..

[71] Vázquez M.E., Blanco J.B., Salvadori S., Trapella C., Argazzi R., Bryant S.D., Jinsmaa Y., Lazarus L.H., Negri L., Giannini E. (2006). 6-N, N-Dimethylamino-2, 3-naphthalimide: A new environment-sensitive fluorescent probe in δ-and μ-selective opioid peptides. J. Med. Chem..

[72] Yu Y., Shao X., Cui Y., Liu H.M., Wang C.L., Fan Y.Z., Liu J., Dong S.L., Cui Y.X., Wang R. (2007). Structure–activity study on the spatial arrangement of the third aromatic ring of endomorphins 1 and 2 using an atypical constrained C terminus. ChemMedChem.

[73] Liu W.X., Wang R. (2012). Endomorphins: Potential roles and therapeutic indications in the development of opioid peptide analgesic drugs. Med. Res. Rev..

[74] Cardillo G., Gentilucci L., Tolomelli A. (2006). Unusual amino acids: Synthesis and introduction into naturally occurring peptides and biologically active analogues. Mini Rev. Med. Chem..

[75] Gentilucci L. (2004). SNew Trends in the Development of Opioid Peptide Analogues as Advanced Remedies for Pain Relief. Curr. Top. Med. Chem..

[76] Liu Z., Udenigwe C.C. (2019). Role of food-derived opioid peptides in the central nervous and gastrointestinal systems. J. Food Biochem..

[77] Kastin A. (2013). Handbook of Biologically Active Peptides.

[78] Yoshikawa M., Takahashi M., Yang S. (2003). Delta opioid peptides derived from plant proteins. Curr. Pharm. Des..

[79] Zioudrou C., Streaty R.A., Klee W.A. (1979). Opioid peptides derived from food proteins. The exorphins. J. Biol. Chem..

[80] Bell S.J., Grochoski G.T., Clarke A.J. (2006). Health implications of milk containing β-casein with the A2 genetic variant. Crit. Rev. Food Sci. Nutr..

[81] Nguyen D.D., Johnson S.K., Busetti F., Solah V.A. (2015). Formation and degradation of beta-casomorphins in dairy processing. Crit. Rev. Food Sci. Nutr..

[82] El-Salam M.A., El-Shibiny S. (2013). Bioactive peptides of buffalo, camel, goat, sheep, mare, and yak milks and milk products. Food Rev. Int..

[83] Brantl V., Teschemacher H. (1979). A material with opioid activity in bovine milk and milk products. Naunyn-Schmiedeberg’s Arch. Pharmacol..

[84] Hazum E., Sabatka J.J., Chang K.-J., Brent D.A., Findlay J., Cuatrecasas P. (1981). Morphine in cow and human milk: Could dietary morphine constitute a ligand for specific morphine (mu) receptors?. Science.

[85] Henschen A., Brantl V., Teschemacher H., Lottspeich F. (1980). β-Casomorphins–Novel Opioid Peptides Derived from Bovine Casein–Isolation and Structure. Endogenous and Exogenous Opiate Agonists and Antagonists.

[86] Loukas S., Varoucha D., Zioudrou C., Streaty R.A., Klee W.A. (1983). Opioid activities and structures of alpha-casein-derived exorphins. Biochemistry.

[87] Brantl V., Teschemacher H., Bläsig J., Henschen A., Lottspeich F. (1981). Opioid activities of β-casomorphins. Life Sci..

[88] Koch G., Wiedemann K., Teschemacher H. (1985). Opioid activities of human β-casomorphins. Naunyn-Schmiedeberg Arch. Pharmacol..

[89] Bouglé D., Bouhallab S. (2017). Dietary bioactive peptides: Human studies. Crit. Rev. Food Sci. Nutr..

[90] Calvo C.F., Cesselin F., Gelman M., Glowinski J. (2000). Identification of an opioid peptide secreted by rat embryonic mixed brain cells as a promoter of macrophage migration. Eur. J. Neurosci..

[91] Lister J., Fletcher P.J., Nobrega J.N., Remington G. (2015). Behavioral effects of food-derived opioid-like peptides in rodents: Implications for schizophrenia?. Pharmacol. Biochem. Behav..

[92] Pruimboom L., De Punder K. (2015). The opioid effects of gluten exorphins: Asymptomatic celiac disease. J. HealthPopul. Nutr..

[93] Ohinata K., Agui S., Yoshikawa M. (2007). Soymorphins, novel μ opioid peptides derived from soy β-conglycinin β-subunit, have anxiolytic activities. Biosci. Biotechnol. Biochem..

[94] Yoshikawa M., Tani F., Yoshimura T., Chiba H. (1986). Opioid peptides from milk proteins. Agric. Biol. Chem..

[95] Brantl V., Pfeiffer A., Herz A., Henschen A., Lottspeich F. (1982). Antinociceptive potencies of β-casomorphin analogs as compared to their affinities towards μ and δ opiate receptor sites in brain and periphery. Peptides.

[96] Tani F., Iio K., Chiba H., Yoshikawa M. (1990). Isolation and characterization of opioid antagonist peptides derived from human lactoferrin. Agric. Biol. Chem..

[97] Sharma S.K., Klee W.A., Nirenberg M. (1975). Dual regulation of adenylate cyclase accounts for narcotic dependence and tolerance. Proc. Natl. Acad. Sci. USA.

[98] Hughes J., Kosterlitz H., Leslie F.M. (1975). Effect of morphine on adrenergic transmission in the mouse vas deferens. Assessment of agonist and antogonist potencies of narcotic analgesics. Br. J. Pharmacol..

[99] Pert C.B., Pasternak G., Snyder S.H. (1973). Opiate agonists and antagonists discriminated by receptor binding in brain. Science.

[100] Yang S., Kawamura Y., Yoshikawa M. (2003). Effect of rubiscolin, a δ opioid peptide derived from Rubisco, on memory consolidation. Peptides.

[101] Kaneko K., Lazarus M., Miyamoto C., Oishi Y., Nagata N., Yang S., Yoshikawa M., Aritake K., Furuyashiki T., Narumiya S. (2012). Orally administered rubiscolin-6, a δ opioid peptide derived from Rubisco, stimulates food intake via leptomeningeal lipocallin-type prostaglandin D synthase in mice. Mol. Nutr. Food Res..

[102] Miyazaki Y., Kaneko K., Iguchi S., Mizushige T., Kanamoto R., Yoshikawa M., Shimizu T., Ohinata K. (2014). Orally administered δ opioid agonist peptide rubiscolin-6 stimulates food intake in aged mice with ghrelin resistance. Mol. Nutr. Food Res..

[103] Hirata H., Sonoda S., Agui S., Yoshida M., Ohinata K., Yoshikawa M. (2007). Rubiscolin-6, a δ opioid peptide derived from spinach Rubisco, has anxiolytic effect via activating σ1 and dopamine D1 receptors. Peptides.

[104] Kaneko K., Mizushige T., Miyazaki Y., Lazarus M., Urade Y., Yoshikawa M., Kanamoto R., Ohinata K. (2014). δ-Opioid receptor activation stimulates normal diet intake but conversely suppresses high-fat diet intake in mice. Am. J. Physiol. Regul. Integr. Comp. Physiol..

[105] Schusdziarra V., Schick A., de la Fuente A., Specht J., Klier M., Brantl V., Pfeiffer E.-F. (1983). Effect of β-casomorphins and analogs on insulin release in dogs. Endocrinology.

[106] Mihatsch W., Franz A., Kuhnt B., Högel J., Pohlandt F. (2005). Hydrolysis of casein accelerates gastrointestinal transit via reduction of opioid receptor agonists released from casein in rats. Neonatology.

[107] Zoghbi S., Trompette A., Claustre J., Homsi M.E., Garzón J., Jourdan G., Scoazec J.-Y., Plaisancié P. (2006). β-Casomorphin-7 regulates the secretion and expression of gastrointestinal mucins through a μ-opioid pathway. Am. J. Physiol. Gastrointest. Liver Physiol..

[108] Yin H., Miao J., Zhang Y. (2010). Protective effect of β-casomorphin-7 on type 1 diabetes rats induced with streptozotocin. Peptides.

[109] Fanciulli G., Dettori A., Demontis M.P., Tomasi P.A., Anania V., Delitala G. (2005). Gluten exorphin B5 stimulates prolactin secretion through opioid receptors located outside the blood-brain barrier. Life Sci..

[110] Belyaeva Y.A., Dubynin V., Stovolosov I., Dobryakova Y.V., Bespalova Z.D., Kamenskii A. (2009). Effects of acute and chronic administration of exorphin C on behavior and learning in white rat pups. Mosc. Univ. Biol. Sci. Bull..

[111] Jinsmaa Y., Yoshikawa M. (1999). Enzymatic release of neocasomorphin and β-casomorphin from bovine β-casein. Peptides.

[112] Fukudome S.-I., Yoshikawa M. (1992). Opioid peptides derived from wheat gluten: Their isolation and characterization. Febs Lett..

[113] Fukudome S.-I., Yoshikawa M. (1993). Gluten exorphin C: A novel opioid peptide derived from wheat gluten. FEBS Lett..

[114] Yang S., Yunden J., Sonoda S., Doyama N., Lipkowski A.W., Kawamura Y., Yoshikawa M. (2001). Rubiscolin, a δ selective opioid peptide derived from plant Rubisco. FEBS Lett..

[115] Matthies H., Stark H., Hartrodt B., Ruethrich H.-L., Spieler H.-T., Barth A., Neubert K. (1984). Derivatives of β-casomorphins with high analgesic potency. Peptides.

[116] Sakaguchi M., Koseki M., Wakamatsu M., Matsumura E. (2006). Effects of systemic administration of β-casomorphin-5 on learning and memory in mice. Eur. J. Pharmacol..

[117] Grecksch G., Schweigert C., Matthies H. (1981). Evidence for analgesic activity of β-casomorphin in rats. Neurosci. Lett..

[118] Lin L., Umahara M., York D., Bray G. (1998). β-Casomorphins stimulate and enterostatin inhibits the intake of dietary fat in rats. Peptides.

[119] Kaneko K., Iwasaki M., Yoshikawa M., Ohinata K. (2010). Orally administered soymorphins, soy-derived opioid peptides, suppress feeding and intestinal transit via gut μ1-receptor coupled to 5-HT1A, D2, and GABAB systems. Am. J. Physiol. Gastrointest. Liver Physiol..

[120] Chesnokova E., Saricheva N., Dubynin V., Kamenskij A., Kalikhevich V., Adermasova Z. (2014). Behavioral effect of soymorphin-5-amide in rats. Mosc. Univ. Biol. Sci. Bull..

[121] Boullin D., Crampton R., Heading C.E., Pelling D. (1973). Intestinal absorption of dipeptides containing glycine, phenylalanine, proline, β-alanine or histidine in the rat. Clin. Sci. Mol. Med..

[122] Matthews D. (1975). Intestinal absorption of peptides. Physiol. Rev..

[123] Gardner M. (1975). Absorption of amino acids and peptides from a complex mixture in the isolated small intestine of the rat. J. Physiol..

[124] Zaloga G.P., Siddiqui R.A. (2004). Biologically active dietary peptides. Mini Rev. Med. Chem..

[125] Kaspar A.A., Reichert J.M. (2013). Future directions for peptide therapeutics development. Drug Discov. Today.

[126] Houston M.E., Campbell A.P., Lix B., Kay C.M., Sykes B.D., Hodges R.S. (1996). Lactam bridge stabilization of α-helices: The role of hydrophobicity in controlling dimeric versus monomeric α-helices. Biochemistry.

[127] Timmerman P., Puijk W.C., Meloen R.H. (2007). Functional reconstruction and synthetic mimicry of a conformational epitope using CLIPS™ technology. J. Mol. Recognit. Interdiscip. J..

[128] Sim S., Kim Y., Kim T., Lim S., Lee M. (2012). Directional assembly of α-helical peptides induced by cyclization. J. Am. Chem. Soc..

[129] Bougatef A., Nedjar-Arroume N., Ravallec-Plé R., Leroy Y., Guillochon D., Barkia A., Nasri M. (2008). Angiotensin I-converting enzyme (ACE) inhibitory activities of sardinelle (Sardinella aurita) by-products protein hydrolysates obtained by treatment with microbial and visceral fish serine proteases. Food Chem..

[130] Chen J., Wang Y., Zhong Q., Wu Y., Xia W. (2012). Purification and characterization of a novel angiotensin-I converting enzyme (ACE) inhibitory peptide derived from enzymatic hydrolysate of grass carp protein. Peptides.

[131] Silvestre M.P.C., Silva M.R., Silva V.D.M., Souza M.W.S.d., Junior L., de Oliveira C., Afonso W.d.O. (2012). Analysis of whey protein hydrolysates: Peptide profile and ACE inhibitory activity. Braz. J. Pharm. Sci..

[132] Darewicz M., Borawska J., Vegarud G.E., Minkiewicz P., Iwaniak A. (2014). Angiotensin I-converting enzyme (ACE) inhibitory activity and ACE inhibitory peptides of salmon (Salmo salar) protein hydrolysates obtained by human and porcine gastrointestinal enzymes. Int. J. Mol. Sci..

[133] Nakamura K., Naramoto K., Koyama M. (2013). Blood-pressure-lowering effect of fermented buckwheat sprouts in spontaneously hypertensive rats. J. Funct. Foods.

[134] García-Tejedor A., Saánchez-Rivera L., Castelló-Ruiz M., Recio I., Salom J.B., Manzanares P. (2014). Novel antihypertensive lactoferrin-derived peptides produced by Kluyveromyces marxianus: Gastrointestinal stability profile and in vivo angiotensin I-converting enzyme (ACE) inhibition. J. Agric. Food Chem..

[135] Ha G.E., Chang O.K., Jo S.-M., Han G.-S., Park B.-Y., Ham J.-S., Jeong S.-G. (2015). Identification of antihypertensive peptides derived from low molecular weight casein hydrolysates generated during fermentation by Bifidobacterium longum KACC 91563. Korean J. Food Sci. Anim. Resour..

[136] Jarmołowska B., Kostyra E., Krawczuk S., Kostyra H. (1999). β-Casomorphin-7 isolated from Brie cheese. J. Sci. Food Agric..

[137] Stuknytė M., Maggioni M., Cattaneo S., De Luca P., Fiorilli A., Ferraretto A., De Noni I. (2015). Release of wheat gluten exorphins A5 and C5 during in vitro gastrointestinal digestion of bread and pasta and their absorption through an in vitro model of intestinal epithelium. Food Res. Int..

[138] De Noni I., Cattaneo S. (2010). Occurrence of β-casomorphins 5 and 7 in commercial dairy products and in their digests following in vitro simulated gastro-intestinal digestion. Food Chem..

[139] Coda R., Rizzello C.G., Pinto D., Gobbetti M. (2012). Selected lactic acid bacteria synthesize antioxidant peptides during sourdough fermentation of cereal flours. Appl. Environ. Microbiol..

[140] Yang R., Zou Y., Yu N., Gu Z. (2011). Accumulation and identification of angiotensin-converting enzyme inhibitory peptides from wheat germ. J. Agric. Food Chem..

[141] Farvin K.S., Baron C.P., Nielsen N.S., Otte J., Jacobsen C. (2010). Antioxidant activity of yoghurt peptides: Part 2–characterisation of peptide fractions. Food Chem..

[142] Pripp A.H., Isaksson T., Stepaniak L., Sørhaug T. (2004). Quantitative structure-activity relationship modelling of ACE-inhibitory peptides derived from milk proteins. Eur. Food Res. Technol..

[143] Wu C.-H., Kuo Y.-H., Hong R.-L., Wu H.-C. (2015). α-Enolase–binding peptide enhances drug delivery efficiency and therapeutic efficacy against colorectal cancer. Sci. Transl. Med..

[144] Wu C.-H., Liu I.-J., Lu R.-M., Wu H.-C. (2016). Advancement and applications of peptide phage display technology in biomedical science. J. Biomed. Sci..

[145] Cavazos A., Gonzalez de Mejia E. (2013). Identification of bioactive peptides from cereal storage proteins and their potential role in prevention of chronic diseases. Compr. Rev. Food Sci. Food Saf..

[146] Udenigwe C.C., Gong M., Wu S. (2013). In silico analysis of the large and small subunits of cereal RuBisCO as precursors of cryptic bioactive peptides. Process Biochem..

[147] Lacroix I.M., Li-Chan E.C. (2012). Evaluation of the potential of dietary proteins as precursors of dipeptidyl peptidase (DPP)-IV inhibitors by an in silico approach. J. Funct. Foods.

[148] Holton T.A., Pollastri G., Shields D.C., Mooney C. (2013). CPPpred: Prediction of cell penetrating peptides. Bioinformatics.

[149] Chandrudu S., Simerska P., Toth I. (2013). Chemical methods for peptide and protein production. Molecules.

[150] Nishiuchi Y., Inui T., Nishio H., BOdi J., Kimura T., Tsuji F.I., Sakakibara S. (1998). Chemical synthesis of the precursor molecule of the Aequorea green fluorescent protein, subsequent folding, and development of fluorescence. Proc. Natl. Acad. Sci. USA.

[151] Carpino L.A., Ghassemi S., Ionescu D., Ismail M., Sadat-Aalaee D., Truran G.A., Mansour E., Siwruk G.A., Eynon J.S., Morgan B. (2003). Rapid, continuous solution-phase peptide synthesis: Application to peptides of pharmaceutical interest. Org. Process Res. Dev..

[152] Murata H., Carmali S., Baker S.L., Matyjaszewski K., Russell A.J. (2018). Solid-phase synthesis of protein-polymers on reversible immobilization supports. Nat. Commun..

[153] Dirksen A., Meijer E., Adriaens W., Hackeng T.M. (2006). Strategy for the synthesis of multivalent peptide-based nonsymmetric dendrimers by native chemical ligation. Chem. Commun..

[154] Lovrinovic M., Niemeyer C.M. (2007). Microtiter plate-based screening for the optimization of DNA–protein conjugate synthesis by means of expressed protein ligation. ChemBioChem.

[155] Hartmann R., Meisel H. (2007). Food-derived peptides with biological activity: From research to food applications. Curr. Opin. Biotechnol..

[156] Meisel H., FitzGerald R.J. (2003). Biofunctional peptides from milk proteins: Mineral binding and cytomodulatory effects. Curr. Pharm. Des..

[157] Mendis E., Rajapakse N., Byun H.-G., Kim S.-K. (2005). Investigation of jumbo squid (Dosidicus gigas) skin gelatin peptides for their in vitro antioxidant effects. Life Sci..

[158] González-García E., Marina M.L., García M.C. (2014). Plum (*Prunus domestica* L.) by-product as a new and cheap source of bioactive peptides: Extraction method and peptides characterization. J. Funct. Foods.

[159] Agyei D., Danquah M.K. (2011). Industrial-scale manufacturing of pharmaceutical-grade bioactive peptides. Biotechnol. Adv..

[160] Kim J., Kim S.-K. (2013). Bioactive peptides from marine sources as potential anti-inflammatory therapeutics. Curr. Protein Pept. Sci..

[161] Udenigwe C.C., Aluko R.E. (2011). Chemometric analysis of the amino acid requirements of antioxidant food protein hydrolysates. Int. J. Mol. Sci..

[162] Ahn J., Park S., Atwal A., Gibbs B., Lee B. (2009). Angiotensin I-converting enzyme (ACE) inhibitory peptides from whey fermented by Lactobacillus species. J. Food Biochem..

[163] Hou P., Zhao G., He C., Wang H., He H. (2018). Biopanning of polypeptides binding to bovine ephemeral fever virus G 1 protein from phage display peptide library. BMC Vet. Res..

[164] Suire C.N., Nainar S., Fazio M., Kreutzer A.G., Paymozd-Yazdi T., Topper C.L., Thompson C.R., Leissring M.A. (2018). Peptidic inhibitors of insulin-degrading enzyme with potential for dermatological applications discovered via phage display. PLoS ONE.

[165] Khondee S., Piyawattanametha W. (2019). Targeting Peptides Derived from Phage Display for Clinical Imaging. Bacteriophages-Biology and Applications.

[166] Shen Y., Ruan L., Lian C., Li R., Tu Z., Liu H. (2019). Discovery of HB-EGF binding peptides and their functional characterization in ovarian cancer cell lines. Cell Death Discov..

[167] Christiansen A., Kringelum J.V., Hansen C.S., Bøgh K.L., Sullivan E., Patel J., Rigby N.M., Eiwegger T., Szépfalusi Z., De Masi F. (2015). High-throughput sequencing enhanced phage display enables the identification of patient-specific epitope motifs in serum. Sci. Rep..

[168] Ramaraju H., Miller S.J., Kohn D.H. (2017). Dual-functioning peptides discovered by phage display increase the magnitude and specificity of BMSC attachment to mineralized biomaterials. Biomaterials.

[169] Yang X., Zhang F., Luo J., Pang J., Yan S., Luo F., Liu J., Wang W., Cui Y., Su X. (2016). A new non-muscle-invasive bladder tumor-homing peptide identified by phage display in vivo. Oncol. Rep..

[170] Fricker L.D. (2012). Neuropeptides and other bioactive peptides: From discovery to function. Colloquium Series on Neuropeptides.

[171] Law P.-Y., Wong Y.H., Loh H.H. (2000). Molecular mechanisms and regulation of opioid receptor signaling. Annu. Rev. Pharmacol. Toxicol..

[172] De Noni I., FitzGerald R.J., Korhonen H.J., Le Roux Y., Livesey C.T., Thorsdottir I., Tomé D., Witkamp R. (2009). Review of the potential health impact of β-casomorphins and related peptides. EFSA Sci. Rep..

[173] Ganapathy V., Miyauchi S. (2005). Transport systems for opioid peptides in mammalian tissues. AAPS J..

[174] Furness J.B., Callaghan B.P., Rivera L.R., Cho H.-J. (2014). The enteric nervous system and gastrointestinal innervation: Integrated local and central control. Microbial Endocrinology: The Microbiota-Gut-Brain Axis in Health and Disease.

[175] Brasnjevic I., Steinbusch H.W., Schmitz C., Martinez-Martinez P., Initiative E.N.R. (2009). Delivery of peptide and protein drugs over the blood–brain barrier. Prog. Neurobiol..

[176] Wang J., Hogenkamp D.J., Tran M., Li W.-Y., Yoshimura R.F., Johnstone T.B., Shen W.-C., Gee K.W. (2006). Reversible lipidization for the oral delivery of leu-enkephalin. J. Drug Target..

[177] Brugos B., Hochhaus G. (2004). Metabolism of dynorphin A (1–13). Die Pharm.-Int. J. Pharm. Sci..

[178] Negri L., Lattanzi R., Tabacco F., Scolaro B., Rocchi R. (1998). Glycodermorphins: Opioid peptides with potent and prolonged analgesic activity and enhanced blood-brain barrier penetration. Br. J. Pharmacol..

[179] Kim B.-J., Zhou J., Martin B., Carlson O.D., Maudsley S., Greig N.H., Mattson M.P., Ladenheim E.E., Wustner J., Turner A. (2010). Transferrin fusion technology: A novel approach to prolonging biological half-life of insulinotropic peptides. J. Pharmacol. Exp. Ther..

[180] Dennis M.S., Zhang M., Meng Y.G., Kadkhodayan M., Kirchhofer D., Combs D., Damico L.A. (2002). Albumin binding as a general strategy for improving the pharmacokinetics of proteins. J. Biol. Chem..

[181] Egleton R.D., Mitchell S.A., Huber J.D., Janders J., Stropova D., Polt R., Yamamura H.I., Hruby V.J., Davis T.P. (2000). Improved bioavailability to the brain of glycosylated Met-enkephalin analogs. Brain Res..

[182] Perlikowska R., Gach K., Fichna J., Toth G., Walkowiak B., do-Rego J.-C., Janecka A. (2009). Biological activity of endomorphin and [Dmt1]endomorphin analogs with six-membered proline surrogates in position 2. Bioorganic Med. Chem..

[183] Tóth G., Keresztes A., Tömböly C., Péter A., Fülöp F., Tourwé D., Navratilova E., Varga É., Roeske W.R., Yamamura H.I. (2004). New endomorphin analogs with mu-agonist and delta-antagonist properties. Pure Appl. Chem..

[184] Bali A., Singh N., Singh Jaggi A. (2014). Neuropeptides as therapeutic targets to combat stress-associated behavioral and neuroendocrinological effects. CNS Neurol. Disord. Drug Targets (Former. Curr. Drug Targets-Cns Neurol. Disord.).

[185] Clynen E., Swijsen A., Raijmakers M., Hoogland G., Rigo J.-M. (2014). Neuropeptides as targets for the development of anticonvulsant drugs. Mol. Neurobiol..

[186] Yimit D., Hoxur P., Amat N., Uchikawa K., Yamaguchi N. (2012). Effects of soybean peptide on immune function, brain function, and neurochemistry in healthy volunteers. Nutrition.

[187] Bernet F., Montel V., Noël B., Dupouy J.P. (2000). Diazepam-like effects of a fish protein hydrolysate (Gabolysat PC60) on stress responsiveness of the rat pituitary-adrenal system and sympathoadrenal activity. Psychopharmacology.

[188] Perlikowska R., Janecka A. (2018). Rubiscolins-highly potent peptides derived from plant proteins. Mini Rev. Med. Chem..

[189] Jahan-Mihan A., Luhovyy B.L., El Khoury D., Anderson G.H. (2011). Dietary proteins as determinants of metabolic and physiologic functions of the gastrointestinal tract. Nutrients.

[190] Yoshikawa M. (2015). Bioactive peptides derived from natural proteins with respect to diversity of their receptors and physiological effects. Peptides.

[191] Booij L., Merens W., Markus C.R., Van der Does A.W. (2006). Diet rich in α-lactalbumin improves memory in unmedicated recovered depressed patients and matched controls. J. Psychopharmacol..

